# The psychological stress response of COVID-19 to medical staff and prevention: A large sample study from China

**DOI:** 10.3389/fpsyg.2023.1125847

**Published:** 2023-03-22

**Authors:** Mei Hu, Yuan Xu, Dengying Bu, Kai Luo, Liying Chang, Chun Mao

**Affiliations:** ^1^Department of Neurology, Xiangyang Central Hospital, Affiliated Hospital of Hubei University of Arts and Science, Xiangyang, China; ^2^Comprehensive Stroke Center, Xiangyang Central Hospital, Affiliated Hospital of Hubei University of Arts and Science, Xiangyang, China; ^3^School of Food Science and Technology and School of Chemical Engineering, Hubei University of Arts and Science, Xiangyang, China; ^4^School of Clinical Medicine, Xiangyang Central Hospital, Affiliated Hospital of Hubei University of Arts and Science, Xiangyang, China

**Keywords:** COVID-19, psychology, stress response, medical workers, clinical work

## Abstract

**Objectives:**

In the face of the COVID-19 pandemic, medical staff in China were more likely to suffer from psychological problems. By investigating the actual state of psychological stress response of medical staff during the COVID-19 outbreak, the study discussed and analyzed the influencing factors of different psychological states in order to prevent the occurrence of serious adverse emotional events in medical staff.

**Methods:**

In the Xiangyang Central Hospital, 1,466 medical staff members have adopted the Psychological Questionnaire for Emergencies Events of Public Health (PQEEPH), which includes questions about depression, neurasthenia, fear, obsessive anxiety, and hypochondriac disorders. The questionnaire also asks about gender, age, education level, health, department, position, and whether personnel exposure history correlation analysis has been confirmed.

**Results:**

The survey revealed that 55% had depression, 26.7% had neurasthenia, 95% had fear, 47.9% had obsessive anxiety, and 69.3% had hypochondria. The effects of depression and hypochondriac emotional stress were significantly greater in female workers than in male workers (*p* < 0.05). Those with higher educational levels had a stronger emotional stress response. Medical professionals with or without contact histories, those who were suspected or confirmed, as well as those in various positions and departments, all demonstrated significant differences in their stress emotions (*p* < 0.05).

**Conclusion:**

Emotional stress affected medical professionals, especially doctors and nurses, who were on the front lines of clinical work in the face of significant public health emergencies. Therefore, to reduce the stress burden and enhance mental health on medical staff, hospitals were suggested to improve their emergency management practices. In addition, the sensitization knowledge training and psychological counseling for front-line clinical staff should be strengthened.

## 1. Introduction

Coronavirus Disease 2019 (COVID-19) is a virus pandemic. 2019-nCoV, a novel coronavirus that formed a clade within the subgenus sarbecovirus, Orthocoronavirinae subfamily, was isolated from human airway epithelial cells using unbiased sequencing to identify a previously unknown B-coronavirus in samples from pneumonia patients (Aslam and Mehra, 2020; Zhu et al., 2020). Many studies in China (Menon et al., 2022), Germany (Stangier et al., 2021), Greece (Golemis et al., 2022), India (Shoib et al., 2021), Italy (Montanaro et al., 2021), Malaysia (Wong and Alias, 2021), Pakistan (Hayat et al., 2021), Spain (Romero-Gonzalez et al., 2021), and the United States of America demonstrate that a significant portion of the general population exhibits significant symptoms of anxiety, depression, and posttraumatic stress (Haikalis et al., 2022).

In December 2019, the Novel Coronavirus outbreak started in Wuhan, Hubei Province, China, and spread around the world with astonishing speed (Chen et al., 2020). The Chinese Centers for Disease Control and Prevention (CCDC) has classified the epidemic as a Class B national infectious disease and has implemented prevention and control measures for Class A infectious diseases because of the epidemic’s ongoing growth (Xiao et al., 2020). The WHO officially recognized the outbreak as a Public Health Emergency of International Concern (PHEIC) on January 31, 2020 (Kang and Xu, 2020; Worsnop et al., 2021).

As of February 24, 2020, a total of 3,387 medical workers from 476 medical institutions in China have been infected with COVID-19, and six people have died, of which 90.4% (3,062 cases) are from Hubei Province (Zhang et al., 2021). The COVID-19 pandemic has had an unprecedented impact on healthcare systems, also has significant and lasting psychological effects on patients and medical workers (Rashid et al., 2021). And it also meant increased family and work obligations for healthcare workers (Feeley et al., 2021). A study revealed that a high proportion of front line healthcare workers had negative emotional response (Kebede et al., 2021; Olivares-Tirado and Zanga-Pizarro, 2022). Strict measures never used in China in modern times were used to contain the virus’ spread. Major cities and entire countries implemented widespread “red zones” and quarantines, advised residents to stay at home, and then instituted lockdown restrictions on incoming and outgoing travelers, on gatherings, and the closure of schools and businesses (Li et al., 2020). Many negative psychosocial effects in nurses have been reported, including stress, anxiety, and depression symptoms caused by fear, high mortality rates, and uncertainty (Kakin et al., 2020). Another study, conducted with 30 Chinese nurses working on the frontlines, identified the causes of negative psychological experiences of nurses during the COVID-19 pandemic as heavy workload, pressure, fear, anxiety, helplessness, and unfamiliarity with the environment and disease (Kandemir et al., 2021). A study examined the social representations of the SARS-CoV-2 virus in Italy, and the most common categories were virus spread, negative feelings, life during quarantine, and virus health consequences (Guarino et al., 2021). Negative lifestyle behaviors were associated with an increased risk of adverse outcomes from coronavirus disease, such as decreased physical activity, decreased sleep, and increased smoking (Radwan et al., 2021). While one-third women lost weight and a significant proportion gained weight during the COVID-19 pandemic in Saudi Arabia, it needed to carefully consider those at risk in the future (Al-Musharaf et al., 2021).

The COVID-19 outbreak presented a significant challenge and stress to frontline medical workers in China, and stress is closely related to a psychological response (Feng and Yin, 2021; Obbarius et al., 2021). The psychological stress response of front-line medical workers providing medical support to COVID-19 patients, on the other hand, was unknown (Boyadzhieva et al., 2020; Kroll et al., 2021). The worst-affected city is Xiangyang, which is adjacent to Wuhan (Yuan et al., 2020). Every day, the Xiangyang Central Hospital, the largest grade-A hospital in the Northwest Hubei province, more than 200 patients with fever were received daily. Working stress may have a direct effect on anxiety and an indirect effect on anxiety through a sense of control (Ripp et al., 2020; Saffari et al., 2021; Hou et al., 2022). As the number of people infected with suspected or confirmed COVID-19 rises, healthcare workers face not only increased workloads but also the risk of infection. Because of the uniqueness and high risk of the job, the psychological strain on all positions in the hospital is enormous, particularly on front-line clinical medical staff such as fever clinic, fever ward, 120 emergency centers, CT room, and other workers who come into contact with fever patients. By understanding the psychological state and characteristics of medical workers in the outbreak of a novel Coronavirus, this study aims to provide a reference for the formulation of a psychological crisis intervention plan in emergency cases.

## 2. Methods

### 2.1. Subjects

From February 6th to March 16th, 2020, a group of people worked at the Xiangyang Central Hospital, the Affiliated Hospital of the Hubei University of Arts and Science in Xiangyang, China.

With an average daily outpatient reception capacity of 6,500 and a medical staff of 4,000, Xiangyang Central Hospital is one of the largest grade-A hospitals in the Northwest Hubei province.

During the COVID-19 pandemic, a random online sampling survey was conducted among the staff of Xiangyang Central Hospital to collect the psychological stress response of medical staff. 2,000 questionnaires were distributed, with 1,466 effective questionnaires recovered at a 73.3% recovery rate. All participants volunteered to participate.

The ethics committees of Xiangyang Central Hospital, the Affiliated Hospital of the Hubei University of Arts and Science in Xiangyang, China, approved the study. Before any study-related procedure, all participants were informed about the investigational nature of the study, the use of their data, and the signed informed consent form.

### 2.2. Research methods

All subjects were questioned and tested in an anonymous online personal interview.

The online questionnaire survey method was used, and questionnaires were distributed to hospital staff anonymously by scanning Quick-Response codes from the questionnaire star platform.^1^ The PQEEPH comprises 25 items divided into five dimensions: depression, neurasthenia, fear, obsessive anxiety, and hypochondria (Huang et al., 2021). The score is determined by the degree of emotional response (none, mild, moderate, and severe). The dimension score is calculated by dividing the total score for each dimension by the number of items. The theoretical maximum and minimum scores are 3 and 0, respectively. The higher a dimension’s score, the more severe the emotional reaction of the subjects on that dimension.

### 2.3. Quality control

(1) Investigators training: The Xiangyang Central Hospital trained neurologists to ensure the consistency and accuracy of the questionnaire. (2) To ensure the authenticity and accuracy of the questionnaire, trained inspectors distributed it online to 2,000 medical workers in the hospital. (3) One-to-one coaching for medical staff who have any questions about filling out the questionnaire, to minimize errors.

### 2.4. Statistical analysis

Comparison of multiple groups of general data: for measurement data, a one-way ANOVA was used, and for multiple rate comparison and statistical hypothesis of count data, Joint Hypotheses Test (*f*-test), or Statistical Hypothesis Test was used. For confidence and significance level, the Student’s *t*-Test (*t*-test) was used to compare the measurement data (both agreed with the normal distribution), the SNK-Q test for pair comparison, and the rank-sum test for non-normal distribution. SPSS 22.0 and Origin 2019b software were used to statistically process all data, and *p* < 0.05 was considered statistically significant. Each value represents the mean of four replicates ± standard deviation (SD).

## 3. Results

### 3.1. General information for medical workers

There were 263 (17.9%) males and 1,203 (82.1%) females among the 1,466 subjects. Age statistics: 1,015 (69.2%, young) were ≤ 44 years old, while 451 (30.8%, middle-aged) were > 44 years old. Education: 314 (21.4%) had a junior college degree or less, 840 (57.3%) had a bachelor degree, and 312 (21.3%) had a master’s degree or higher. There were 396 doctors (27.0%), 854 nurses (58.3%), 173 technicians (11.8%), and 43 administrative personnel (2.9%). COVID-19 patients’ contact history: 885 (60.4%) had no contact history, while 581 (39.6%) had contact history. Their COVID-19 infection status was as follows: 1,440 non-COVID-19 patients (98.2%) and 26 COVID-19 patients (1.8%). Departments: 174 (11.9%) in the fever outpatient department and fever ward, 119 (8.1%) in the emergency center and ED (emergency department), 448 (30.6%) in the clinical medical department, 448 (30.5%) in the internal medicine department and ward, 234 (16.0%) in the surgical department and ward, and 43 (2.9%) in the administrative department.

**Figure 1:**
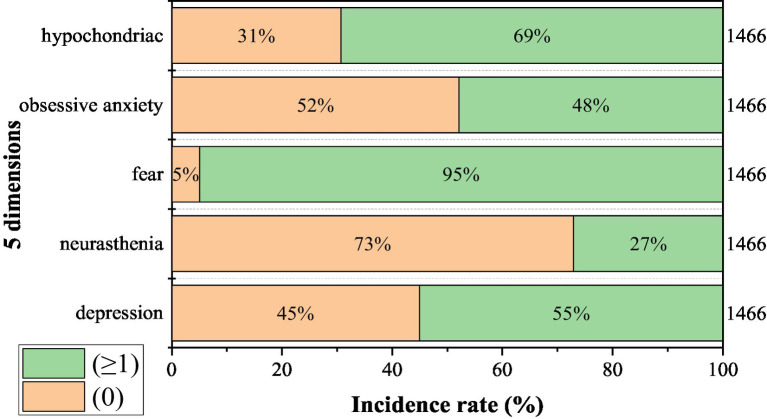
Incidence of five dimensions in 1466 subjects (n, %).

Figure 1 depicts the incidence of the five dimensions of the subjects, where 0 indicated emotional stress did not affect them and ≥ 1 indicated emotional stress affected them. In 1,466 subjects, the incidence of five dimensions were depression 807, accounting for 55%, neurasthenia 397, accounting for 26.7%, fear 1,392, accounting for 95%, obsessive anxiety 702, 47.9%, and hypochondria 1,016, accounting for 69%.

### 3.2. Emotional stress responses in different gender and age groups

Depression and hypochondria were significantly different in gender (*p* < 0.05), with female medical workers scoring 2.82 ± 2.42, slightly higher than males scoring 1.81 ± 2.53. Female medical workers scored 1.35 ± 1.26, slightly higher than males scoring 1.21 ± 1.26. We found women to be more susceptible to depression, hypochondria, and other stress responses than men. In terms of age, there was no significant difference between the two groups of age ≤ 40 and > 40 but at 40 (*p* > 0.05; Table 1).

**Table 1:** Comparison of emotional dimensions in different genders and age groups.

Projects	Male (*n* = 263)	Female (*n* = 1,203)	*T*	*p*	Age ≤ 40 (*n* = 1,131)	Age > 40 (*n* = 335)	*T*	*p*
Depression	1.81 ± 2.53	2.82 ± 2.42	0.044	0.034	1.82 ± 2.46	1.80 ± 2.38	0.135	0.892
Neurasthenia	2.72 ± 3.01	2.74 ± 2.85	0.601	0.082	2.73 ± 2.89	2.77 ± 2.85	0.235	0.814
Fear	4.34 ± 3.44	4.48 ± 3.25	1.680	0.093	4.43 ± 3.25	4.56 ± 3.41	0.627	0.531
Obsessive anxiety	1.28 ± 2.16	1.35 ± 2.09	0.076	0.060	1.33 ± 2.09	1.37 ± 2.18	0.357	0.721
Hypochondriac	1.21 ± 1.26	1.35 ± 1.26	0.440	0.036	1.33 ± 1.26	1.32 ± 1.28	0.174	0.862

Each value represents the mean of four replicates ± standard deviation (SD).

### 3.3. Comparison of emotional stress response to health status and novel coronavirus exposure

Besides obsessive anxiety, there were significant differences in the four dimensions of depression, neurasthenia, fear, and hypochondriasis between the infected and non-infected persons (*p* < 0.05). The scores of the infected in all five dimensions were higher than those of the uninfected persons (Table 2), indicating that the emotional stress response of COVID-19 infected persons was stronger than that of healthy persons.

**Table 2:** Comparison of differences in emotional dimensions of one’s health status and whether or not there is a novel Coronavirus exposure history.

Projects	Healthy (*n* = 1,440)	Infected (*n* = 26)	*T*	*p*	Non-contact (*n* = 1,131)	Contact (*n* = 335)	*T*	*p*
Depression	1.78 ± 2.38	3.81 ± 4.26	4.228	0.023	1.59 ± 2.30	2.15 ± 2.59	4.329	0.001
Neurasthenia	2.69 ± 2.83	5.39 ± 4.43	4.767	0.042	2.46 ± 2.75	3.14 ± 3.02	4.423	0.040
Fear	4.40 ± 3.22	7.81 ± 4.94	5.297	0.020	4.14 ± 3.1371	4.93 ± 3.44	4.546	0.012
Obsessive anxiety	1.29 ± 2.01	3.50 ± 4.54	5.330	0.210	1.06 ± 1.82	1.75 ± 2.42	6.163	0.009
Hypochondriac	1.31 ± 1.24	2.15 ± 1.75	3.389	0.022	1.20 ± 1.22	1.49 ± 1.31	4.324	0.041

Each value represents the mean of four replicates ± standard deviation (SD).

In terms of contact history (Table 2), there were significant differences in the five dimensions between the two groups (*p* < 0.05). The scores for depression without contact history were 1.59 ± 2.30, while the scores for contact history were 2.15 ± 2.59, the scores for obsessive anxiety without contact history were 1.06 ± 1.82, and the score of contact history was 1.75 ± 2.42, the difference was extremely significant (*p* < 0.01). This demonstrated that exposure to novel coronavirus-infected patients adversely affected every aspect of the psychological and emotional health of medical personnel.

### 3.4. Comparison of emotional stress responses under different educational levels

The results of the F-test for different educational levels showed that there was no significant difference between the bachelor group and the postgraduate or above group (*p* > 0.05), but there were differences in all five dimensions compared with the college or below group (*p* < 0.05), and the higher the educational level, the stronger the emotional stress response (Table 3). There were extremely significant differences between the two dimensions of neurasthenia and obsessive anxiety (*p* < 0.01).

**Table 3:** Comparison of emotional dimensions of medical staff under different educational levels and different positions.

Projects	College or below (*n* = 314)	Bachelor (*n* = 840)	Postgraduate or above (*n* = 312)	*F*	*p*	Doctor (*n* = 1,440)	Nurse (*n* = 26)	Technician (*n* = 1,131)	Administration (*n* = 335)	*F*	*p*
Depression	1.62 ± 2.29	1.92 ± 2.20	1.73 ± 2.39	1.835	0.011	1.78 ± 2.33	1.92 ± 2.52	1.50 ± 2.34	1.23 ± 1.76	2.313	0.027
Neurasthenia	2.55 ± 2.74	2.84 ± 2.91	2.63 ± 2.95	0.469	0.008	2.78 ± 2.93	2.83 ± 2.89	2.25 ± 2.71	2.73 ± 2.88	4.274	0.041
Fear	4.39 ± 3.16	4.53 ± 3.29	4.33 ± 3.40	3.534	0.035	4.43 ± 3.44	4.64 ± 3.25	3.76 ± 2.99	3.67 ± 3.01	8.553	0.025
Obsessive anxiety	1.13 ± 1.92	1.41 ± 2.11	1.36 ± 2.28	1.435	0.009	1.35 ± 2.17	1.40 ± 2.13	1.12 ± 1.98	0.70 ± 2.88	2.388	0.008
Hypochondriac	1.29 ± 1.17	1.39 ± 1.29	1.17 ± 1.25	2.019	0.033	1.17 ± 1.23	1.45 ± 1.28	1.09 ± 1.16	0.90 ± 1.01	2.164	0.042

Each value represents the mean of four replicates ± standard deviation (SD).

### 3.5. Comparison of emotional stress response of medical staff in different posts

An F-test of medical staff members in various positions revealed that the doctor and nurse groups significantly outperformed the technician and administration groups in five dimensions (*p* < 0.05), with an extremely significant difference in obsessive anxiety (*p* < 0.01; Table 3).

### 3.6. Comparison of emotional stress response of medical staff in different departments

Depression, neurasthenia, and hypochondria were significantly different between departments (*p* < 0.05). The clinical medical department and fever outpatient department had the highest depression dimension scores, with 2.12 ± 2.40 and 2.04 ± 2.69, respectively. The highest scores in the neurasthenia dimension were obtained by the emergency department and the internal medicine department, which were 2.96 ± 2.81 and 2.87 ± 2.91, respectively. The fever outpatient department and the internal medicine department had the highest hypochondriasis dimension scores, with 1.43 ± 1.36 and 1.41 ± 1.23 points, respectively (Table 4).

**Table 4:** Comparison of emotional dimensions of staff in different departments.

Projects	Fever outpatient department (*n* = 174)	Emergency department (*n* = 119)	Clinical medical department (*n* = 448)	Internal medicine department (*n* = 448)	Surgical department (*n* = 234)	Administrative department (*n* = 43)	*F*	*p*
depression	2.04 ± 2.69	1.65 ± 2.32	2.12 ± 2.40	1.89 ± 2.44	1.60 ± 2.38	1.94 ± 2.51	1.432	0.013
neurasthenia	2.74 ± 2.90	2.96 ± 2.81	2.57 ± 2.79	2.87 ± 2.91	2.50 ± 2.84	2.81 ± 2.95	2.362	0.013
fear	4.26 ± 3.30	4.25 ± 3.19	4.37 ± 2.84	4.76 ± 3.39	4.35 ± 3.25	3.79 ± 3.10	2.313	0.076
obsessive anxiety	1.24 ± 2.09	1.16 ± 2.01	1.38 ± 2.15	1.58 ± 2.25	1.09 ± 1.72	1.27 ± 1.99	1.307	0.058
hypochondriac	1.43 ± 1.36	1.19 ± 1.23	1.39 ± 1.15	1.41 ± 1.23	1.25 ± 1.27	1.16 ± 1.23	3.546	0.010

Each value represents the mean of four replicates ± standard deviation (SD).

## 4. Discussion

Fear was prevalent in this study (95%), which could be attributed to the fact that the cluster infection had been reported among the medical staff in many hospitals in Wuhan as well as the disease’s limited understanding and the lack of effective medical measures or vaccine intervention in the early stages of the outbreak. Medical staff was primarily concerned about their families and themselves were infected, so they washed their hands repeatedly, scrub surfaces and appeared paranoid. Depression and hypochondriasis were prevalent in 55 and 69.3% of the population, respectively, and were primarily manifested as a slow reaction, insomnia, easy fatigue, and difficulty in concentration. They were concerned about the hospital’s limited protective materials and suspected that their workplace prevention and control procedures were not standardized, such as lack of strict hand hygiene and disinfection and lack of a standard mask tightness test. When removing protective clothing, they are especially concerned about touching clean clothes inside and spreading infection. According to some studies on the impact of the SARS outbreak on social psychology, approximately 20% of people were depressed, and the incidence rate of depression among nurses was 45% (Maunder et al., 2006), which was like the incidence rate of depression in this study. The SARS outbreak’s impact on people is primarily manifested in sleep disorders, which include difficulty falling asleep, difficulty maintaining sleep, and decreased sleep satisfaction (Elser et al., 2021). Compared with this study, during the COVID-19 pandemic, medical staff suffered psychological fear, neurasthenia and other problems, which also caused different degrees of damage to their sleep quality.

### 4.1. Emotional stress response of medical workers of different genders

In this study, female medical workers’ emotional stress reaction was higher than males’; because nurses are mostly female (854 nurses, including 22 males and 832 females). The study also discovered that nurses with various jobs showed significant differences in the five dimensions of emotional stress reaction, with many recent studies on psychological research showing that nurses during the outbreak reported similar findings (Abdulah et al., 2021; Walecka et al., 2021). The first reason is that the outbreak is sudden and highly infectious, and many infected clinical nurses are under great psychological pressure. Secondly, because most nurses had not participated in systematic disaster emergency training or public health emergencies, they lacked experience dealing with unexpected infectious diseases. Thirdly, nurses have extensive contact with patients as well as a heavy workload and pressure. Many medical procedures, such as venous blood collection, infusion, vital sign monitoring, and so on, necessitate close contact between nurses and patients; even minor errors can cause infection (Ottum et al., 2013). The inconvenience of wearing protective clothing (such as airtight, blurred vision, and inflexible hands after wearing gloves) caused heavy difficulties for the actual operation of nurses during the COVID-19 lockdown period.

### 4.2. Emotional stress responses to COVID-19 infection

In this study, 26 medical workers were diagnosed with COVID-19, and the intensity of their emotional stress response was greater than that of healthy medical workers. There were significant differences between the two groups in depression, fear, and hypochondria (*p* < 0.05). The intensity of the five dimensions of emotional stress response was higher in medical workers with a history of contact with infected people than in medical workers without a history of contact (*p* < 0.05). The following were the primary causes of the psychological stress reaction: (1) Novel Coronavirus hypochondriasis is a psychological condition caused by other disease symptoms, as well as a suspicion that the current symptoms of physical discomfort are caused by the novel Coronavirus. (2) Since the outbreak of the epidemic, the number of infected people across the country has risen sharply because of environmental factors, the maximum emergency response mechanism has been activated, and the entire nation has entered a state of war readiness. People who have been infected with the virus must be isolated for treatment, and anyone who has had contact with them (including family members, friends, and even strangers) must be sent to isolation sites regardless of clinical symptoms. Medical personnel were prone to anxiety and fear in such an environment. (3) In the early stages of the epidemic, a lack of understanding of the virus, as well as unreliable public opinion information and rumors, exacerbated several psychological issues, such as sensitivity, suspicion, and fear of clinical workers (Mohammed, 2021; Tindle et al., 2022).

### 4.3. Emotional stress response of medical staff in different posts and departments

During the SARS epidemic, some researchers studied the mental health of medical staff and discovered that there were significant differences in depression and anxiety among the medical staff in different departments (Moeller et al., 2021; Onchonga et al., 2021). Medical staff in high-risk environments had poorer mental health than those in low-risk environments, and they were nervous and lacked coping skills (Engelbrecht et al., 2021; Tolksdorf et al., 2022). The emotional stress response of medical staff in direct contact with COVID-19 patients, such as fever outpatient and ward, medical technology department (CT room, laboratory department), and emergency department, was found to be more significant than in general medicine, surgery, and functional departments (*p* < 0.05), according to this study. The following are some explanations: (1) At the beginning of the epidemic, many patients flooded into Xiangyang’s emergency department and fever clinic. To assist, the hospital mobilized much medical personnel from the department of non-respiratory and infectious diseases. It was natural to be nervous when meeting critically ill patients because of a lack of experience. Even experienced doctors and nurses can feel helpless in the face of death. The medical staff has seen many such tragic scenes, and psychological overload will appear on the face of the death of patients without relatives to see them off (Sun et al., 2020; Omolola et al., 2021; Tolksdorf et al., 2022). The term vicarious trauma (VT) refers to witnessing many cruel and destructive scenes, the impact of which exceeds some people’s psychological and emotional tolerance, resulting in a variety of psychological abnormalities. These abnormal phenomena, usually motivated by sympathy and empathy for the survivors and their trauma, cause serious physical and mental distress if not complete mental breakdown. According to studies, vicarious trauma is caused primarily by the personal factors of rescuers and specific environmental factors (Fernandes et al., 2021; Lankarani et al., 2021). (2) The fever clinic and ward are in different departments of the hospital, and the interaction between medical staff requires mutual understanding, support, and tolerance. Different working styles, team cultures, interpersonal pressure, and medical personnel will all cause physical and mental problems.

Based on the experience of previous SARS or Ebola outbreaks (Jefferies et al., 2020; Keshvardoost et al., 2020), and in light of the COVID-19 pandemic, the following suggestions were made to improve the psychological condition of medical staff: (1) To reduce the risk of novel coronavirus infection, hospitals can improve infection control knowledge training for medical staff and develop relevant management procedures and emergency plans. (2) Make human resource allocation and protective material scheduling more scientific and reasonable to ensure the orderly development of logistics work and reduce clinical medical workers’ psychological stress. (3) Improve medical staff’s sense of vocation and belonging through humanistic care and encouragement. (4) Understand the psychological dynamics of medical workers on time, focus on physical and mental health, and open online and offline psychological clinics for consultation and counseling regularly. (5) Update epidemic information, reduce medical staff panic, and alleviate psychological fear and anxiety.

This study has some limitations due to the online random sampling survey of medical staff. Despite the ongoing COVID-19 pandemic, future research would continue to focus on the mental health of medical staff in hospitals and expand the scope of investigations to conduct face-to-face research.

## 5. Conclusion

Based on a comprehensive analysis of a sample of 1,466 hospital workers, the study found that clinical doctors, nurses and management staff were all affected by both physical and psychological factors during the COVID-19 pandemic, which resulted in varying levels of depression, neurasthenia, fear, obsessive anxiety and stress responses. Therefore, to reduce the stress burden and enhance mental health on medical staff, hospitals were suggested to improve their emergency management practices. In addition, the sensitization knowledge training and psychological counseling for front-line clinical staff should be strengthened, and to help health staff better prepare for the possibility of another pandemic in the future.

## Data availability statement

The original contributions presented in the study are included in the article/supplementary material; further inquiries can be directed to the corresponding authors.

## Ethics statement

The studies involving human participants were reviewed and approved by Institutional Review Board at the Xiangyang Central Hospital, the Affiliated Hospital of Hubei University of Arts and Science. The patients/participants provided their written informed consent to participate in this study.

## Author contributions

MH, YX, KL, and LC contributed to the concept and design of this research and the writing and revised the article many times. MH, YX, DB, KL, and CM contributed to this method and the collection of cases. MH, YX, and DB contributed to the statistical analysis. MH, YX, DB, KL, LC, and CM have made substantial contributions to the manuscript and accept responsibility for the content of the manuscript. All authors contributed to the article and approved the submitted version.

## Funding

This work was supported by Xiangyang Science and Technology project (grant number 2021ABA003624), Science and Technology support action plan of the Hubei Provincial Department of Education (grant number BXLBX0693), and National Natural Science Foundation of China (grant number 42207525).

## Conflict of interest

The authors declare that the research was conducted in the absence of any commercial or financial relationships that could be construed as a potential conflict of interest.

## Publisher’s note

All claims expressed in this article are solely those of the authors and do not necessarily represent those of their affiliated organizations, or those of the publisher, the editors and the reviewers. Any product that may be evaluated in this article, or claim that may be made by its manufacturer, is not guaranteed or endorsed by the publisher.

## Footnotes

^1^ www.wjx.cn

## References

[ref1] Abdulah D. M. Abdulla B. M. O. Liamputtong P. (2021). Psychological response of children to home confinement during COVID-19: a qualitative arts-based research. Int. J. Soc. Psychiatry 67, 761–769. doi: 10.1177/0020764020972439, PMID: 33183155

[ref2] Al-Musharaf S. Aljuraiban G. Bogis R. Alnafisah R. Aldhwayan M. Tahrani A. (2021). Lifestyle changes associated with COVID-19 quarantine among young Saudi women: a prospective study. PLoS One 16:e0250625. doi: 10.1371/journal.pone.0250625, PMID: 33914800 PMC8084143

[ref3] Aslam S. Mehra M. R. (2020). COVID-19: yet another coronavirus challenge in transplantation. J. Heart Lung Transplant. 39, 408–409. doi: 10.1016/j.healun.2020.03.007, PMID: 32253113 PMC7141445

[ref4] Boyadzhieva V. V. Stoilov N. R. Stoilov R. M. (2020). Coronavirus disease 2019 (COVID-19) during pregnancy in patients with rheumatic diseases. Rheumatol. Int. 40, 1753–1762. doi: 10.1007/s00296-020-04698-y, PMID: 32930863 PMC7490482

[ref5] Chen W. Horby P. W. Hayden F. G. Gao G. F. (2020). A novel coronavirus outbreak of global health concern. Lancet 395, 470–473. doi: 10.1016/S0140-6736(20)30185-9, PMID: 31986257 PMC7135038

[ref7] Elser H. Kiang M. V. John E. M. Simard J. F. Bondy M. Nelson L. M.. (2021). The impact of the first COVID-19 shelter-in-place announcement on social distancing, difficulty in daily activities, and levels of concern in the San Francisco Bay Area: a cross-sectional social media survey. PLoS One 16:e0244819. doi: 10.1371/journal.pone.0244819, PMID: 33444363 PMC7808609

[ref8] Engelbrecht M. C. Heunis J. C. Kigozi N. G. (2021). Post-traumatic stress and coping strategies of south African nurses during the second wave of the COVID-19 pandemic. Int. J. Environ. Res. Public Health 18:7919. doi: 10.3390/IJERPH18157919, PMID: 34360211 PMC8345364

[ref9] Feeley T. Ffrench-O’Carroll R. Tan M. H. Magner C. O’Connor E. (2021). A model for occupational stress amongst paediatric and adult critical care staff during COVID-19 pandemic. Int. Arch. Occup. Environ. Health 94, 1721–1737. doi: 10.1007/s00420-021-01670-6, PMID: 33630134 PMC7905984

[ref10] Feng L. Yin R. (2021). Social support and Hope mediate the relationship between gratitude and depression among front-line medical staff during the pandemic of COVID-19. Front. Psychol. 12:623873. doi: 10.3389/fpsyg.2021.623873, PMID: 33776846 PMC7987792

[ref11] Fernandes N. Costa D. Costa D. Keating J. Arantes J. (2021). Predicting COVID-19 vaccination intention: the determinants of vaccine hesitancy. Vaccine 9, 1161–1177. doi: 10.3390/VACCINES9101161, PMID: 34696269 PMC8538665

[ref12] Golemis A. Voitsidis P. Parlapani E. Nikopoulou V. A. Tsipropoulou V. Karamouzi P.. (2022). Young adults’ coping strategies against loneliness during the COVID-19-related quarantine in Greece. Health Promot. Int. 37, 1–13. doi: 10.1093/heapro/daab053, PMID: 33864073 PMC8138818

[ref13] Guarino A. Prati G. Barbieri I. Cicognani E. Tzankova I. Compare C.. (2021). Peoples understanding of the COVID-19 pandemic: social representations of SARS-CoV-2 virus in Italy. Health Risk Soc. 23, 304–320. doi: 10.1080/13698575.2021.1972089

[ref14] Haikalis M. Doucette H. Meisel M. K. Birch K. Barnett N. P. (2022). Changes in college student anxiety and depression from pre- to during-COVID-19: perceived stress, academic challenges, loneliness, and positive perceptions. Emerg. Adulthood 10, 534–545. doi: 10.1177/21676968211058516, PMID: 35382515 PMC8919103

[ref15] Hayat K. Haq M. Wang W. Khan F. U. Fang Y. (2021). Impact of the COVID-19 outbreak on mental health status and associated factors among general population: a cross-sectional study from Pakistan. Psychol. Health 27, 54–68. doi: 10.1080/13548506.2021.1884274, PMID: 33627000

[ref16] Hou Y. Hou W. Zhang Y. Liu W. Chen A. (2022). Relationship between working stress and anxiety of medical workers in the COVID-19 situation: a moderated mediation model. J. Affect. Disord. 297, 314–320. doi: 10.1016/j.jad.2021.10.072, PMID: 34715184 PMC8556767

[ref17] Huang Z. Zhang L. Wang J. Xu L. Li Y. Guo M.. (2021). The structural characteristics and influential factors of psychological stress of urban residents in Jiangxi province during the COVID-19 pandemic: cross sectional study. Heliyon 7:e07829. doi: 10.1016/j.heliyon.2021.e07829, PMID: 34485727 PMC8405985

[ref18] Jefferies M. Rashid H. Hill-Cawthorne G. Kayser V. (2020). A brief history of ebolavirus disease: paving the way forward by learning from the previous outbreaks. Infect. Dis. Drug Targets 20, 259–266. doi: 10.2174/1871526518666181001125106, PMID: 30277167

[ref19] Kakin Z. Iydem E. Aci Z. S. Kutlu Y. (2020). Experiences and psychosocial problems of nurses caring for patients diagnosed with COVID-19 in Turkey: a qualitative study. Int. J. Soc. Psychiatry 67, 158–167. doi: 10.1177/0020764020942788, PMID: 32674644

[ref20] Kandemir D. Ylmaz A. Snmez B. (2021). Professional and psychological perceptions of emergency nurses during the COVID-19 pandemic: a qualitative study. Jpn. J. Nurs. Sci. 19:e12470. doi: 10.1111/jjns.12470, PMID: 34970852

[ref21] Kang Y. Xu S. (2020). Comprehensive overview of COVID based on current evidence. Dermatol. Ther. 33:e13525. doi: 10.1111/dth.13525, PMID: 32378801 PMC7267400

[ref22] Kebede M. A. Demissie D. B. Guddu D. K. Haile M. T. Muleta M. B. (2021). Emotional responses and perceived stressors of frontline medical staffs in case of COVID-19 treatment centers and obstetrics emergency in Ethiopia. BMC Psychiatry 21:308. doi: 10.1186/s12888-021-03311-1, PMID: 34130631 PMC8204075

[ref23] Keshvardoost S. Bahaadinbeigy K. Fatehi F. (2020). Role of telehealth in the management of COVID-19: lessons learned from previous SARS, MERS, and Ebola outbreaks. Telemed. e-health 26, 850–852. doi: 10.1089/tmj.2020.0105, PMID: 32329659

[ref24] Kroll K. H. Larsen S. Lamb K. Davies W. H. Apps J. N. (2021). Responding to the psychological needs of health-care workers during the COVID-19 pandemic: case study from the Medical College of Wisconsin. J. Clin. Psychol. Med. 29, 150–161. doi: 10.1007/s10880-021-09791-3, PMID: 34059975 PMC8166374

[ref25] Lankarani K. B. Honarvar B. Sadati A. K. Haghighi M. R. R. (2021). Citizens’ opinion on governmental response to COVID-19 outbreak: a qualitative study from Iran. INQUIRY: the journal of health care organization. Provis. Financ. 58, 1–11. doi: 10.1177/00469580211024906, PMID: 34166135 PMC8239969

[ref26] Li X. Wang W. Zhao X. Zai J. Zhao Q. Li Y.. (2020). Transmission dynamics and evolutionary history of 2019-nCoV. J. Med. Virol. 92, 501–511. doi: 10.1002/jmv.25701, PMID: 32027035 PMC7166881

[ref27] Maunder R. Lancee W. Balderson K. Bennett J. Borgundvaag B. Evans S.. (2006). Long-term psychological and occupational effects of providing hospital healthcare during SARS outbreak. Emerg. Infect. Dis. 12, 1924–1932. doi: 10.3201/eid1212.060584, PMID: 17326946 PMC3291360

[ref28] Menon V. Varadharajan N. Andrade C. (2022). Anxiety and depression in Covid-19 frontline health care workers in China. Int. J. Soc. Psychiatry 68:223. doi: 10.1177/0020764021989744 33478331

[ref29] Moeller A. L. Mills E. H. A. Collatz Christensen H. Gnesin F. Blomberg S. N. F. N. Zylyftari N.. (2021). Symptom presentation of SARS-CoV-2-positive and negative patients: a nested case-control study among patients calling the emergency medical service and medical helpline. BMJ Open 11:e044208. doi: 10.1136/bmjopen-2020-044208, PMID: 34031110 PMC8149264

[ref30] Mohammed A. M. (2021). Sleep disturbance among frontline nurses during the COVID-19 pandemic. Sleep Biol. Rhythms 19, 467–473. doi: 10.1007/s41105-021-00337-6, PMID: 34230810 PMC8247106

[ref31] Montanaro E. Artusi C. A. Rosano C. Boschetto C. Imbalzano G. Romagnolo A.. (2021). Anxiety, depression, and worries in advanced Parkinson disease during COVID-19 pandemic. Neurol. Sci. 43, 341–348. doi: 10.1007/s10072-021-05286-z, PMID: 33948763 PMC8096160

[ref32] Obbarius N. Fischer F. Liegl G. Obbarius A. Rose M. (2021). A modified version of the transactional stress concept according to Lazarus and Folkman was confirmed in a psychosomatic inpatient sample. Front. Psychol. 12:584333. doi: 10.3389/fpsyg.2021.584333, PMID: 33746820 PMC7973375

[ref33] Olivares-Tirado P. Zanga-Pizarro R. (2022). Impact of COVID-19 pandemic outbreak on mental health of the hospital front-line healthcare workers in Chile: a difference-in-differences approach. J. Public Health 8, 1–8. doi: 10.1093/pubmed/fdac008, PMID: 35137226 PMC8903375

[ref34] Omolola A. Daniel H. Kendra S. Luz H. Han D. Lechauncy W. (2021). Correlates of social isolation among minority older adults during the COVID-19 pandemic. Innov. Aging 5, 293–295. doi: 10.1093/geroni/igab046.1137

[ref35] Onchonga D. Ngetich E. Makunda W. Wainaina P. Viktoria P. (2021). Anxiety and depression due to 2019 SARS-CoV-2 among frontier healthcare Workers in Kenya. Heliyon 7:e06351. doi: 10.1016/j.heliyon.2021.e06351, PMID: 33644428 PMC7901492

[ref36] Ottum A. Sethi A. K. Jacobs E. Zerbel S. Gaines M. E. Safdar N. (2013). Engaging patients in the prevention of health care-associated infections: a survey of patients’ awareness, knowledge, and perceptions regarding the risks and consequences of infection with methicillin-resistant Staphylococcus aureus and clostridium diffici. Am. J. Infect. Control 41, 322–326. doi: 10.1016/j.ajic.2012.04.334, PMID: 22884494

[ref37] Radwan H. Kitbi M. A. Hasan H. Hilali M. A. Fjijoer N. (2021). Indirect health effects of COVID-19: unhealthy lifestyle behaviors during the lockdown in the United Arab Emirates. Int. J. Environ. Res. Public Health 18:1964. doi: 10.3390/ijerph18041964, PMID: 33670510 PMC7922937

[ref38] Rashid S. Reeder C. Sahu S. Rashid S. (2021). Psychological distress and moral injury to oncologists and their patients during COVID-19 pandemic. Curr. Psychol. 41, 8175–8180. doi: 10.1007/s12144-021-02128-1, PMID: 34341650 PMC8318552

[ref39] Ripp J. Peccoralo L. Charney D. (2020). Attending to the emotional well-being of the health care workforce in a new York City health system during the COVID-19 pandemic. Acad. Med. 95, 1136–1139. doi: 10.1097/ACM.0000000000003414, PMID: 32282344 PMC7176260

[ref40] Romero-Gonzalez B. Puertas-Gonzalez J. A. Mario-Narvaez C. Peralta-Ramirez M. I. (2021). COVID-19 lockdown variables predicting anxiety and depressive symptoms in pregnant women. Med. Clin. 156, 172–176. doi: 10.1016/j.medcle.2020.10.010, PMID: 33243419 PMC7832526

[ref41] Saffari M. Bashar F. R. Vahedian-Azimi A. Pourhoseingholi M. A. Karimi L. Shamsizadeh M.. (2021). Effect of a multistage educational skill-based program on Nurse’s stress and anxiety in the intensive care setting: a randomized controlled trial. Hindawi 2021:8811347. doi: 10.1155/2021/8811347, PMID: 33986878 PMC8093071

[ref42] Shoib S. Saleem M. Islam M. S. Arafat S. Joseph S. J. (2021). Severity of depression, anxiety and stress among the people of Kashmir, India during COVID-19: an observation from tele psychiatric services. Glob. Psychiatry Arch. 4, 62–67. doi: 10.52095/gp.2021.8115

[ref43] Stangier U. Kananian S. Schüller J. (2021). Perceived vulnerability to disease, knowledge about COVID-19, and changes in preventive behavior during lockdown in a German convenience sample. Curr. Psychol. 41, 7362–7370. doi: 10.1007/s12144-021-01456-6, PMID: 33654348 PMC7906828

[ref44] Sun N. Laiyou L. I. Chen S. Yang S. Liu X. (2020). Status of and factors influencing the anxiety and depression of front-line medical staff supporting Wuhan in containing COVID-19. Jpn. J. Nurs. Sci. 18:e12398. doi: 10.21203/rs.3.rs-19665/v1 33258559 PMC7744847

[ref45] Tindle R. Hemi A. Moustafa A. A. (2022). Social support, psychological flexibility and coping mediate the association between COVID-19 related stress exposure and psychological distress. Sci. Rep. 12:8688. doi: 10.1038/s41598-022-12262-w, PMID: 35606392 PMC9126245

[ref46] Tolksdorf K. H. Tischler U. Heinrichs K. (2022). Correlates of turnover intention among nursing staff in the COVID-19 pandemic: a systematic review. BMC Nurs. 21:174. doi: 10.1186/s12912-022-00949-4, PMID: 35787700 PMC9252069

[ref47] Walecka I. Ciechanowicz P. Dopytalska K. Mikucka-Wituszyńska A. Szymańska E. Bogucki J.. (2021). Psychological consequences of hospital isolation during the COVID-19 pandemic - research on the sample of polish firefighting academy students. Curr. Psychol. 1-10, 1–10. doi: 10.1007/S12144-021-01982-3, PMID: 34220176 PMC8238034

[ref48] Wong L. P. Alias H. (2021). Temporal changes in psychobehavioural responses during the early phase of the COVID-19 pandemic in Malaysia. J. Behav. Med. 44, 18–28. doi: 10.1007/s10865-020-00172-z, PMID: 32757088 PMC7405711

[ref49] Worsnop C. Z. Kamradt-Scott A. Lee K. Grépin K. Rothery F. (2021). Legal compliance is not enough: cross-border travel and trade measures and COVID-19. Int. Stud. Rev. 23, 302–345. doi: 10.1093/isr/viab004

[ref50] Xiao S. Cheng G. Yang R. Zhang Y. Ding Y. (2020). Prediction on the number of confirmed Covid-19 with the FUDAN-CCDC mathematical model and its epidemiology, clinical manifestations, and prevention and treatment effects. Results Physics 20:103618. doi: 10.1016/j.rinp.2020.103618, PMID: 33262927 PMC7687494

[ref51] Yuan H. Liu J. Gao Z. Hu F. (2020). Clinical features and outcomes of acute kidney injury in patients infected with COVID-19 in Xiangyang. Blood Purif. 50, 513–519. doi: 10.1159/000513163, PMID: 33316799 PMC7801975

[ref52] Zhang X. Jiang Y. Yu H. Jiang Y. Li D. (2021). Psychological and occupational impact on healthcare workers and its associated factors during the COVID-19 outbreak in China. Int. Arch. Occup. Environ. 94, 1441–1453. doi: 10.1007/s00420-021-01657-3, PMID: 33656572 PMC7926194

[ref53] Zhu N. Zhang D. Wang W. Li X. Tan W. (2020). A novel coronavirus from patients with pneumonia in China, 2019. N. Engl. J. Med. 382, 727–733. doi: 10.1056/NEJMoa2001017, PMID: 31978945 PMC7092803

